# The impact of chalazion after treatment on the morphology of the meibomian glands in children: An observational study

**DOI:** 10.1097/MD.0000000000048806

**Published:** 2026-05-08

**Authors:** Jiao Jiang, Xiaoge Yang, Feifan Du, Wei Zheng, Yang Yang

**Affiliations:** a Department of Ophthalmology, Hebei Children’s Hospital, Shijiazhuang, Hebei, China; b Department of Ophthalmology, Hebei Clinical Medicine Research Center for Children’s Health and Diseases, Shijiazhuang, Hebei, China; c Department of Abdominal Ultrasonography, The Second Hospital of Hebei Medical University, Shijiazhuang, Hebei, China.

**Keywords:** chalazion, dry eye, meibomian gland dysfunction, meibomian gland loss, ocular surface comprehensive analyzer

## Abstract

Morphological changes in the meibomian glands following treatment of pediatric chalazion were assessed using an ocular surface analyzer, and the effects of surgical versus conservative management on gland morphology were systematically compared. Methods: A total of 132 children with chalazion who had achieved complete recovery after treatment were selected as the research subjects and divided into 2 groups according to different treatment methods: the surgical treatment group (52 cases) and the drug treatment group (80 cases). Meanwhile, 40 asymptomatic healthy children during the same period were selected as the control group. The degree of meibomian gland loss was calculated and scored by using the ocular surface comprehensive analyzer. Result: The meibomian gland loss score in the surgical group was (0.89 ± 0.96), in the drug treatment group it was (0.78 ± 0.61), and in the control group it was (0.40 ± 0.35). There was a significant difference in the score of meibomian gland loss between the chalazion group and the control group (*P* <.05). However, there was no significant difference in the degree of meibomian gland loss between surgical group and the drug group (*P* >.05). Conclusion: Chalazion can lead to meibomian gland loss, and changing the treatment method will not improve this situation. How to control the recurrence of chalazion is particularly important in preventing pediatric meibomian gland dysfunction.

## 1. Introduction

Chalazion are a very common eyelid disease in ophthalmology. The common treatment methods include surgical removal and conservative treatment. The conservative treatment usually involves instilling antibiotic eye drops, combined with hot compress or physical therapy.^[1]^ Both surgical removal and conservative treatment of chalazion will result in superficial scars at the meibomian gland site.^[2]^ The atrophy and deformation of the meibomian gland morphology are the key indicators determining the severity of meibomian gland dysfunction in children. Therefore, our study aims to investigate whether the superficial scars formed after the regression of meibomian gland cysts will affect the function of the meibomian glands. The ocular surface comprehensive analyzer is a noninvasive, noncontact device used for ocular surface evaluation. It enables objective assessment of meibomian gland morphology and structural integrity through optical imaging. Our study aims to evaluate meibomian gland atrophy in pediatric patients with chalazion using this device, and to compare the impact of different treatment modalities on their meibomian gland function.

## 2. Object and methods

### 2.1. Research object and group

Our study employed a prospective research design. A total of 132 children diagnosed with and cured of chalazion in Hebei Children’s Hospital from January 2022 to June 2025 were selected. Cysts are managed on the basis of their size: smaller cysts can be relieved via conservative measures such as antibiotic eye drops and local hot compresses, while larger or recurrent cysts may require surgical treatment to address the condition thoroughly. Physicians provide treatment recommendations to parents based on this principle, allowing them to choose the appropriate therapeutic approach according to their preferences. The patients were divided into 2 groups based on different treatment methods: the surgical treatment group (52 cases) and the drug treatment group (80 cases). The surgery involved performing a general anesthesia procedure to remove the meibomian gland cyst. The cyst was incised and the contents and necrotic tissues within the cyst were scraped away. Then, the surgical cavity was burned using 5% povidone iodine cotton pads. Drug treatment involves applying to the affected eye a local dose of tobramycin eye drops for anti-infection (one drop each time, 3 times a day), combined with local hot compress on the eyelid of the affected eye. If both eyes are affected, the right eye will be selected as the research subject; for a single eye affected, the affected eye is selected as the research subject. One month after the recovery of chalazion, all cases underwent examination of meibomian gland morphology using Dry Eye Analyzer.

Inclusion criteria: meets the diagnostic criteria for chalazion; has no history of wearing contact lenses; no other diseases that may cause abnormal morphology of the meibomian glands have been detected in the eyes; No other autoimmune diseases that may cause dry eye syndrome; Children aged 3 to 15 who can undergo the meibomian gland imaging examination.

Forty children who underwent routine vision examinations at the ophthalmology department during the same period were selected as the control group. The right eye was selected for the evaluation of meibomian gland scores. None of them had a history of chalazion, nor did they present any symptoms or related signs.

Our study was approved by the Ethics Committee of Hebei Children’s Hospital (202136), which complies with the requirements of the Helsinki Declaration. The parents of the children were informed of and agreed to participate in this study, and signed the informed consent form.

### 2.2. Inspection methods

**Figure 1.**
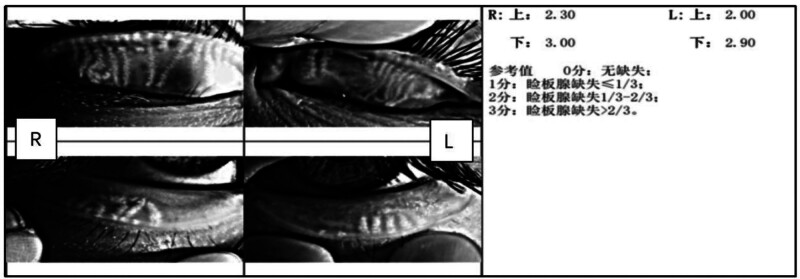
Score for the degree of meibomian gland loss.

Dry Eye Analyzer (Chongqing Kanghua Ruiming Science Technology Co., Ltd): Based on the principle of Placido ring and optical imaging, the morphology of the meibomian glands is objectively evaluated, and the degree of meibomian gland loss is scored (Fig. 1): 0 grade: no loss of meibomian glands; 1 point: meibomian glands loss ≤ 1/3; 2 points: meibomian gland loss from 1/3 to 2/3; 3 points: meibomian gland loss > 2/3. All the examinations were conducted by the same technician. During the examination, the meibomian glands of the upper and lower eyelids need to be fully exposed. Each examination required 3 repetitions, and the average value was recorded. The sum of the scores for the upper eyelid and the lower eyelid represents the score for the degree of meibomian gland deficiency of that eye.

### 2.3. Statistical analysis

The analysis was conducted using SPSS 26.0. The gender counts of each group were expressed as n (%) and the comparisons between groups were performed using the Pearson χ^2^ test. The quantitative data of age, disease duration and meibomian gland loss score were presented as mean ± standard deviation, and the comparisons among the experimental group and the control group were conducted using analysis of variance and least significant difference. *P* <.05 indicated a statistically significant difference.

## 3. Results

### 3.1. Baseline data (age, gender and disease duration)

The surgical group consisted of 52 cases (23 males and 29 females, mean age 4.23 ± 1.71 years, mean duration 7.85 ± 4.53 weeks); the drug group included 80 cases (44 males and 36 females, mean age 4.06 ± 2.03 years, mean duration 10.02 ± 8.65 weeks); the control group had 40 cases (22 males and 18 females, mean age 4.65 ± 1.75 years). There was no statistically significant difference in age, gender and disease duration among the groups (*P* >.05) (Table 1).

**Table 1:** Comparison of differences in age and gender among the 3 groups.

	Surgical group	Drug group	Control group	χ2	*P*
Age (year)	4.23 ± 1.71	4.06 ± 2.03	4.65 ± 1.75	–	.271
Female	29 (55.77%)	36 (45.00%)	18 (45.00%)	1.685	.431
Male	23 (44.23%)	44 (55.00%)	22 (55.00%)		
Duration (week)	7.85 ± 4.53	10.02 ± 8.65	–	–	.115

### 3.2. Comparison of the degree of meibomian gland loss among the surgical group, drug group and control group

**Figure 2.**
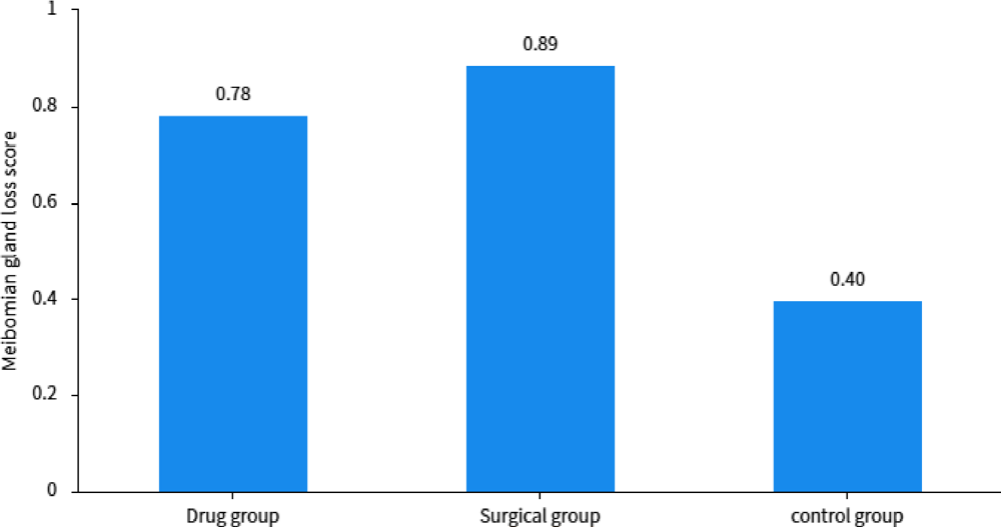
The degree of meibomian gland loss in the 3 groups of patients.

The meibomian gland loss score in the surgical group was (0.89 ± 0.96), in the drug treatment group it was (0.78 ± 0.61), and in the control group it was (0.40 ± 0.35) (Fig. 2). There was a significant difference in the score of meibomian gland loss between the experimental group and the control group (*P* <.05), but there was no significant difference in the degree of meibomian gland loss between the surgical group and the drug group (*P* >.05) (Table 2).

**Table 2:** The differences in the scores of meibomian gland loss among 3 groups.

Group	Number	Score	*F*	*P*	LSD
Surgical group	52	0.89 ± 0.96	6.176	.003	Surgical group, drug group > control group
Drug group	80	0.78 ± 0.61			
Control group	40	0.40 ± 0.35			

LSD = least significant difference.

## 4. Discussion

Chalazion is a chronic nonsuppurative inflammatory granuloma formed when the ducts of the meibomian glands become blocked and the secretions accumulate within the glands.^[3]^ The common treatment methods include surgical treatment and local drug therapy.^[2]^ Both treatment methods have their own advantages and disadvantages. Surgical treatment can directly remove the cyst in a relatively short period of time, especially for some complex types such as those with cysts close to the lacrimal system and eyelid margin.^[4]^ However, surgeries for pediatric patients need to be performed under general anesthesia. Children and their parents have to endure considerable psychological pressure. If there is a recurrence of chalazion after the surgery, it is often very difficult to decide whether to undergo surgical treatment again. Drug therapy is a common conservative treatment method for pediatric chalazion. It is often combined with local hot compress on the eyelid to promote the absorption of the cysts.^[5]^ The onset of chalazion does not cause any pain or other discomfort and does not affect normal eye use, If there is local infection of the cyst, antibiotic eye drops can be applied for eye drops to fight infection. The changes of chalazion may include: the cyst gradually shrinks or gradually enlarges; the cyst ruptures and forms a granuloma; and in most cases of chalazion in children, it belongs to the latter type. The local granuloma further absorbs and forms a local superficial scar.^[6]^

The superficial scars formed during the healing process of chalazion cause abnormal expression of the meibomian gland morphology, which is closely related to the occurrence of meibomian gland dysfunction.^[7]^ Meibomian gland imaging technology enables the visualization of the structure of the meibomian glands, and many scholars have applied it in the research of meibomian glands, objectively evaluating the degree of meibomian gland deficiency.^[8–10]^ The research conducted by Anna Machalińska et al^[11]^ has previously demonstrated that chalazion may be one of the risk factors for the absence of meibomian glands. In our study, the optical imaging of meibomian glands in the ocular surface comprehensive analyzer was applied, which could clearly present and record the degree of meibomian gland loss. Our research results indicated that the score of meibomian gland loss in children with meibomian gland cysts was significantly higher than that in the control group. It is consistent with the research results of Li et al.^[12]^ That is to say, children with chalazion do have varying degrees of meibomian gland loss after recovery. However, under the 2 different treatment methods of surgical treatment and drug treatment, there was no significant difference in the degree of meibomian gland loss in children with chalazion.

The research conducted by Minji Ha et al^[13,14]^ revealed that the absence of meibomian glands does not lead to any changes in the properties of meibomian gland secretions, but it can lead to irregularities at the eyelid margins, congestion and swelling, as well as blockage of the meibomian gland openings, resulting in a reduction in lipid secretion by the meibomian glands. Jun Feng et al research^[15]^ also demonstrated that the abnormal morphology of meibomian glands can adversely affect the function of the meibomian glands. The absence of meibomian glands can cause blockage of the meibomian gland openings. Based on our research results, the healing of pediatric meibomian gland cysts is prone to cause an increase in meibomian gland absence, which is bound to have an adverse impact on the function of the meibomian glands in children and increase the risk of meibomian gland dysfunction.

Our study still has several limitations. This experiment lacks long-term follow-up data for the patients. At the same time, the sample size of this study is small, and there may be biases caused by multiple factors. Therefore, the research results still need to be further verified through large-sample, randomized controlled, multi-center prospective studies. We need to conduct further long-term follow-up for patients with chalazion to observe the morphology of the meibomian glands, the thickness of the lipid layer, the tear film breakup time, and the inflammatory factors of the conjunctiva, and to compare them with those of healthy individuals. This was done to further study the impact of meibomian gland cysts on the ocular surface microenvironment.

## 5. Conclusions

In conclusion, chalazion may lead to the meibomian glands loss, and changing the treatment method will not improve this situation. Therefore, how to control the recurrence of chalazion is particularly important in preventing pediatric meibomian gland dysfunction. We should focus on the medical history of chalazion, combine symptomatic treatment and etiological treatment, attach importance to regular follow-up and public health education, and formulate individualized treatment plans.

## Author contributions

**Conceptualization:** Jiao Jiang, Wei Zheng, Yang Yang.

**Data curation:** Jiao Jiang, Xiaoge Yang, Feifan Du, Wei Zheng, Yang Yang.

**Formal analysis:** Jiao Jiang, Xiaoge Yang.

**Funding acquisition:** Jiao Jiang.

**Investigation:** Feifan Du.

**Methodology:** Jiao Jiang.

**Software:** Yang Yang.

**Supervision:** Yang Yang.

**Writing – original draft:** Yang Yang.

**Writing – review & editing:** Yang Yang.
